# Genome-wide screen for modifiers of Parkinson's disease genes in *Drosophila*

**DOI:** 10.1186/1756-6606-4-17

**Published:** 2011-04-19

**Authors:** Caroline Fernandes, Yong Rao

**Affiliations:** 1 Department of Biology, McGill University Health Centre, 1650 Cedar Avenue, Montreal, Quebec H3G 1A4, Canada; 2 McGill Centre for Research in Neuroscience, Department of Neurology and Neurosurgery, Department of Medicine, McGill University Health Centre, 1650 Cedar Avenue, Montreal, Quebec H3G 1A4, Canada

## Abstract

**Background:**

Mutations in *parkin *and *PTEN-induced kinase 1 *(*Pink1) *lead to autosomal recessive forms of Parkinson's disease (PD). *parkin *and *Pink1 *encode a ubiquitin-protein ligase and a mitochondrially localized serine/threonine kinase, respectively. Recent studies have implicated Parkin and Pink1 in a common and evolutionarily conserved pathway for protecting mitochondrial integrity.

**Results:**

To systematically identify novel components of the PD pathways, we generated a genetic background that allowed us to perform a genome-wide F1 screen for modifiers of *Drosophila parkin *(*park*) and *Pink1 *mutant phenotype. From screening ~80% of the fly genome, we identified a number of cytological regions that interact with *park *and/or *Pink1*. Among them, four cytological regions were selected for identifying corresponding PD-interacting genes. By analyzing smaller *deficiency *chromosomes, available transgenic RNAi lines, and P-element insertions, we identified five PD-interacting genes. Among them, *opa1 *and *drp1 *have been previously implicated in the PD pathways, whereas *debra (dbr), Pi3K21B *and *β4GalNAcTA *are novel PD-interacting genes.

**Conclusions:**

We took an unbiased genetic approach to systematically isolate modifiers of PD genes in *Drosophila*. Further study of novel PD-interacting genes will shed new light on the function of PD genes and help in the development of new therapeutic strategies for treating Parkinson's disease.

## Background

Parkinson's disease (PD) is the second most common neurodegenerative disease. It is characterized by the loss of nigral dopaminergic neurons. Mutations in *Pink1 *and *Parkin *cause autosomal recessive early-onset Parkinson's disease in humans [1,2]. Together mutations in these genes account for greater than 50% of familial Parkinson disease (PD) and ~20% of early-onset sporadic cases [3-5]. Recent studies on characterizing the function of Parkin and Pink1 have significantly advanced our understanding of PD pathogenesis.

Parkin has E3-ubiquitin ligase activity, and is shown to degrade abnormally folded proteins [6]. For instance, Parkin ubiquitinates and degrades proteins such as CDCrel-1 [7], Parkin-associated endothelin receptor-like (Pael) receptor [8], α-synuclein [9], synphilin-1 [10], and cyclin E [11]. Thus, Parkin dysfunction in regulating the level of other proteins or itself through protein degradation may contribute to PD pathogenesis.

Pink1 is a mitochondria-localized serine/threonine kinase [2,12,13]. A recent study suggests that Pink1 directly phosphorylates Parkin [14]. In addition, Pink1 may directly or indirectly induce the phosphorylation of the HSP75 chaperone TRAP1 [12] and the mitochondrial protease HtrA2 [13].

Accumulated evidence supports that Pink1 and Parkin act together in a common and conserved pathway to protect mitochondrial integrity (for review, see [15]). For instance, it is reported that overexpression of *Drosophila Parkin *(*park*) could rescue mitochondrial defects caused by *Pink1 *mutations both in *Drosophila *[16-19] and mammalian systems [20,21]. Recent studies also indicate that Pink1-dependent recruitment of Park into mitochondria is required for the clearance of damaged mitochondria [22-25].

*Drosophila melanogaster *has proven to be a powerful model system for understanding the function of PD genes. Several PD genes such as *park*, *Pink1*, *LRRK2 *and *HtrA1 *have orthologs in *Drosophila*. Interfering with their function caused PD-like phenotypes in *Drosophila *[17-19,26-29]. Genetic studies in *Drosophila *have begun to reveal new targets for the development of new therapeutic approaches to treat PD. For instance, Pallanck and colleagues previously conducted a genetic screen to isolate modifiers of partial lethality caused by complete loss of *park *in *Drosophila *[30]. From ~1400 P-element insertions affecting less than 10% of the fly genome, they identified several genes that regulate oxidative stress and innate immune responses [30].

In this study, we conducted a systematic genetic screen to isolate *park*- and/or *Pink1*-interacting regions that cover ~80% of the entire fly genome. We generated a genetic background in which *park *or *Pink1 *was knocked down. The availability of this genetic tool allowed us to perform a F1 genetic screen to identify cytological regions on the 2^nd ^and 3^rd ^chromosome that interact with *park *and/or *Pink1*. We found that 31 cytological regions modify both *park *and *Pink1 *wing-posture phenotype. In addition, 21 cytological regions showed interactions with both *Pink1 *and *park *in adult lethality test. We then selected four cytological regions for fine mapping, which identified two known PD-interacting genes *opa1 *and *drp1*, and three novel PD-interacting genes *debra, Pi3K21B *and *β4GalNAcTA*.

## Methods

### *Drosophila *stocks

*UAS*-*Pink1-RNAi*, *UAS*-*park RNAi *and other transgenic RNAi lines were obtained from the VDRC stock center. A collection of *deficiencies *uncovering >92% of cytological regions on 2^nd ^and 3^rd ^chromosomes were obtained from the Bloomington *Drosophila *Stock Center. Smaller *deficiencies *and P-element insertions mapped within large PD-interacting cytological regions were also obtained from the Bloomington *Drosophila *Stock Center. Balancer stocks *CyO,GAL80 *and *TM3,GAL80 *were provided by D.van Meyel. The *park*^edpkΔ21^/*TM3,Sb *line was provided by M. Guo (UCLA). *Pink1*^B9^/*FM7,Act-GFP *and *park*^25^/*TM3,Sb *stocks were provided by T. Fon. *Df(2R)β4GalNAcTA*^[20.1] ^and *β4GalNAcTA*^4.1 ^lines were obtained from N. Haines.

### Genetics

To knock down *Pink1 *or *park*, *tubulin-GAL4 *(*tub-GAL4*) flies were crossed with *UAS-Pink1-RNAi *or *UAS-park-RNAi *flies to ubiquitously express *Pink1*-RNAi or *park *RNAi. Since fly stocks with ubiquitous expression of *Pink1-RNAi *or *park RNAi *under control of *tub-GAL4 *are not healthy, genetic crosses were performed to generate *UAS*-*Pink1-RNAi*/*CyO,GAL80; tub-GAL4/TM3,Sb *and *UAS*-*park-RNAi*;*tub-Gal4/TM3,Sb,GAL80 *stocks, in which GAL4 is inhibited by GAL80 to prevent the expression of *UAS-Pink1-RNAi *or *UAS-park-RNAi *in parental stocks [31].

F1 screen was performed by crossing individual *deficiency *lines from 2^nd ^and 3^rd ^chromosome *deficiency *kits with *UAS*-*Pink1-RNAi*/*CyO,GAL80; tub-GAL4/TM3,Sb *or *UAS*-*park-RNAi*;*tub-Gal4/TM3,Sb,GAL80 *flies. The F1 progeny in *Pink1-RNAi *background were reared at 25°, and the F1 progeny in *park-RNAi *background were kept at 29°. F1 progeny were collected for 4-6 days and separated according to their date of eclosion. The modification of wing-posture phenotype by each *deficiency *chromosome was scored on post-eclosion day 3 for *Pink1 *screen and on post-eclosion day 6 for *park *screen. Wing posture phenotype in both male and female F1 flies was scored, and the modifying effect on penetrance was determined by counting the percentage of both held-up-wing flies and drooped-wing flies. For *park *and *Pink1 *screen, 212 and 217 *deficiencies *in the *deficiency *kit were screened, respectively.

Selected *deficiency *lines were also crossed with *Pink1*^B9^/*FM7,Act-GFP *female flies. F1 progeny were scored for the modification of the wing-posture phenotype. The F1 progeny were also scored for adult lethality test.

### Analysis of wing phenotype, longevity and fertility

For analysis of abnormal wing phenotype, ~20 flies were placed per vial. Flies with both wings held-up or drooped were counted.

For longevity test, flies were collected upon eclosion and transferred to new vials every 4-6 days. Mortality was scored daily. The assay was performed in triplicate. Survival curves were plotted using GraphPad software.

To test fertility of male flies, individual male flies were crossed with three (*w1118*) virgin females. After 10 days, the number of vials with progeny were counted.

### Statistical Analysis

Student's t-test was used for statistical analysis.

## Results

### Characterization of *park *and *Pink1 *knockdown phenotypes

Previous studies show that loss of *park *or loss of *Pink1 *caused similar phenotypes, such as abnormal wing morphology, male sterility, reduced climbing ability, decreased longevity and loss of dopaminergic neurons [17-19,29]. To generate a "*park*-inhibited" or "*Pink1-*inhibited" background suitable for systematic F1 genetic screen, we used the *GAL4-UAS *system [32] to knock down the level of *Pink1 *or *park *in flies.

**Figure 1:**
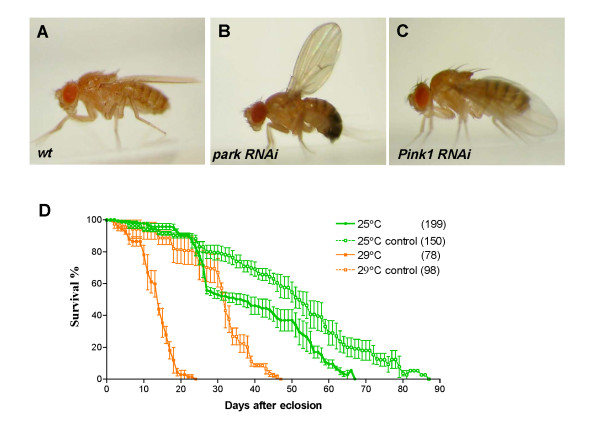
**Knockdown of PD genes induces a wing position phenotype in adult flies**. A-C, Ubiquitous knockdown of PD genes in flies induces an abnormal wing position phenotype (i.e. held up or drooped wing). A. Wild-type wing position in flies carrying only the *tub-GAL4 *driver. B, A *park *knockdown fly (i.e. a fly carrying both the *tub-GAL4 *driver and *UAS-park-RNAi *transgene). The "held-up" wing position phenotype was observed when *UAS-park-RNAi *was ubiquitously expressed under control of the *tub-GAL4 *driver. C, The "drooped" wing phenotype was observed in a *Pink1 *knockdown fly (i.e. a fly carrying both the *tub-GAL4 *driver and *UAS-Pink1-RNAi *transgene). D, Survival curves of *park *knockdown flies (solid line) and control flies (i.e. flies carrying only the *UAS-park *transgene) (dashed line). The experiments were performed at 25°C (green) and 29°C (red). Numbers in brackets represent sample numbers. Error bars represent SEM.

Consistent with previous reports [17-19,29], we found that ubiquitous knockdown of *Pink1 *or *park *by expressing *UAS-park-RNAi *or *UAS-Pink1-RNAi *transgenes under control of the *tub-GAL4 *driver, caused male sterility (compared to 100% fertility in wild-type control, *Pink1 *and *park *knockdown flies showed 14.3% and 44.4% fertility, respectively), reduced life span, and abnormal wing posture (i.e. held up or drooped) (Figure 1). Those phenotypes resembled that observed in *park *and *Pink1 *loss-of-function mutants [17-19,29].

We then tested if the penetrance and severity of above phenotypes could be enhanced by increasing the expression level of the *UAS-park-RNAi *transgene. This was achieved by elevating temperature, which increases the activity of GAL4 leading to higher expression of *UAS-transgenes *[32]. Indeed, we found that increasing the expression level of *park-RNAi *significantly enhanced the phenotype. The penetrance of wing-posture phenotype in *park *knockdown flies was increased from ~2.1% at 25°C to ~22.4% at 29°C. The maximal life span of *park *knockdown flies was further reduced from ~67 days at 25°C to ~17 days at 29°C. The fertility of male *park *knockdown flies was also reduced from ~44.4% at 25°C to ~30% at 29°C.

We also examined the effect of increasing the level of *Pink1-RNAi *transgene on wing posture, male sterility and longevity. In *Pink1 *knockdown flies, the penetrance of wing-posture phenotype was increased from ~2.9% at room temperature to ~91% at 29°C. The maximal life span of *Pink1 *knockdown flies was reduced from ~55 days at room temperature to ~18 days at 29°C. The fertility of male *Pink1 *knockdown flies was also decreased from ~14.3% at room temperature to 0% at 29°C.

### F1 screen for modifiers of the *park *knockdown phenotype

**Figure 2:**
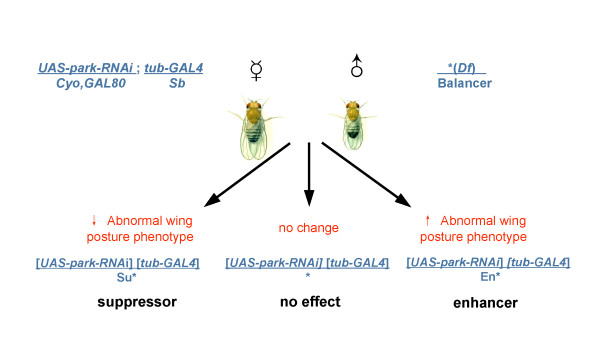
**A genetic scheme for isolating modifiers of Parkinson's disease genes in *Drosophila***. *Deficiencies (Df) *from the Bloomington *deficiency *kits were crossed individually into the *park *or *Pink1 *knockdown mutant background. F1 progeny were scored for potential phenotypic enhancement (i.e. an increase in the penetrance of the wing phenotype) or suppression (i.e. a decrease in the penetrance of the wing phenotype). Abbreviations: En, enhancement; Su, suppression; *tub-GAL4*, *tubulin-GAL4.*

To identify novel modifiers of the PD pathway, we set out to conduct a systematic screen to identify cytological regions on the 2^nd ^and 3^rd ^chromosome that interact with *park *(Figure 2).

Prior to the screen, we examined if the *park *knockdown mutant background is sensitive to the reduction in the dosage of known genes in the pathway. We found that reducing the level of endogenous *park *substantially increased the penetrance of the *park-RNAi*-induced wing posture phenotype from ~15% (n = 76) (genotype: *park RNAi; +/+*) to ~43% (n = 97) (genotype: *park RNAi; park*^25^*/+*) (P < 0.05). Since *Pink1 *and *park *have previously been shown to act in a common pathway [17-19], we also tested if the *park *knockdown background is sensitive to a reduction in the level of *Pink1*. Indeed, we found that *Pink1 *heterozygostiy significantly enhanced the penetrance of the *park-RNAi*-induced wing posture phenotype from ~13% (n = 90) (genotype: *+/+; park RNAi*) to ~40% (n = 32) (genotype: *Pink1*^*B9*^*/+; park RNAi*) (P < 0.01).

To systematically identify modifiers of this *park *wing-posture phenotype, we crossed a large collection of *deficiencies *on the 2^nd ^and 3^rd ^chromosome into the *park *knockdown mutant background. In each *deficiency *chromosome, a portion of cytological regions was deleted. Thus, crossing a *deficiency *chromosome into the *park *knockdown background led to 50% reduction in the dosage of genes located within the deleted cytological region.

From this screen, we identified 26 cytological regions that enhanced the *park *wing-posture phenotype (Table 1), and 53 cytological regions that suppressed the wing-posture phenotype (Table 2). We also found that reducing the dosage of genes by 50% in 48 cytological regions in *park *knockdown flies caused lethality prior to the adult stage (Table 3). No such adult lethality was observed when *park *was knocked down alone, or the dosage of those 48 cytological regions was reduced by 50% in wild type background.

**Table 1:** Enhancers of the *park-RNAi *wing phenotype

*Deficiencies*	Breakpoints	**Strength of modification **^**a**^
*Df(2L)net-PMF*	21A1;21B7-8	++
*Df(2L)BSC28*	23C5-D1;23E2	++++
*Df(2L)cl-h3*	25D2-4;26B2-5	++
*Df(2L)BSC7*	26D10-E1;27C1	++
*Df(2L)ED611*	29B4;29C3	++
*Df(2L)BSC17*	30C3-5;30F1	++
*Df(2L)Mdh*	30D-30F;31F	+++++
*Df(2L)BSC50*	30F5;31B1	++++++
*Df(2L)FCK-20*	32D1;32F1-3	+++
*Df(2R)nap9*	42A1-2;42E6-F1	+++++
*Df(2R)cn9*	42E;44C	++
*Df(2R)H3E1*	44D1-4;44F12	+++
*Df(2R)en30*	48A3-4;48C6-8	+++
*Df(2R)BSC39*	48C5-D1;48D5-E1	++++
*Df(2R)BSC161*	54B2;54B17	++++
*Df(2R)Exel7162*	56F11;56F16	++++
*Df(2R)59AD*	59A1-3;59D1-4	+++
*Df(3L)AC1*	67A2;67D11-13	+++++
*Df(3L)XS533*	76B4;77B	++
*Df(3L)BSC249*	79B2;79D2	++
*Df(3R)BSC47*	83B7-C1;83C6-D1	++
*Df(3R)Tpl10*	83C1-2;84B1-2	++
*Df(3R)BSC43*	92F7-93A1;93B3-6	++
*Df(3R)BSC56*	94E1-2;94F1-2	++
*Df(3R)BSC137*	95A2-4;95A8-B1	+++
*Df(3R)BSC42*	98B1-2;98B3-5	++

Each *deficiency *was crossed into the *park RNA*i background and the wing posture phenotype was scored. Crosses were maintained at 29°C.

^a ^Each '+' represents 1.0 SD from the mean pentrance (i.e. ~22.4%) observed for *park RNAi *alone flies.

**Table 2:** Suppressors of the *park-RNAi *wing phenotype

*Deficiencies*	Breakpoints	**Strength of modification**^**a**^
*Df(2L)BSC106*	21B7;21C2	++++
*Df(2L)dp-79b*	22A2-3;22D5-E1	++++
*Df(2L)ed1*	24A2;24D4	++++
*Df(2L)sc19-8*	24C2-8;25C8-9	++
*Df(2L)BSC110*	25C1;25C4	++
*Df(2L)BSC109*	25C4;25C8	++++
*Df(2L)E110*	25F3-26A1;26D3-11	++
*Df(2L)BSC142*	28C3;28D3	++++
*Df(2L)BSC143*	31B1;31D9	++
*Df(2L)BSC145*	32C1;32C1	+++
*Df(2L)b87e25*	34B12-C1;35B10-C1	++++
*Df(2L)C'*	h35;h38L	++
*Df(2R)BSC3*	48E12-F4;49A11-B6	++++
*Df(2R)Exel7131*	50E4;50F6	++++
*Df(2R)BSC550*	53C1;53C6	++
*Df(2R)robl-c*	54B17-C4;54C1-4	++
*Df(2R)BSC45*	54C8-D1;54E2-7	++++
*Df(2R)P34*	55E2-4;56C1-11	++++
*Df(2R)AA21*	56F9-17;57D11-12	++
*Df(2R)BSC155*	60B9;60C4	+++
*Df(2R)M60E*	60E2-3;60E11-12	++
*Df(3L)Aprt-1*	62A10-B1;62D2-5	++++
*Df(3L)BSC181*	62A11;62B7	++++
*Df(3L)XDI98*	65A2;65E1	++
*Df(3L)BSC33*	65E10-F1;65F2-6	++
*Df(3L)pbl-X1*	65F3;66B10	++++
*Df(3L)66C-G28*	66B8-9;66C9-10	+++
*Df(3L)h-i22*	66D10-11;66E1-2	+++
*Df(3L)Scf-R6*	66E1-6;66F1-6	++
*Df(3L)BSC283*	67C7;67D5	+++
*Df(3L)eyg[C1]*	69A4-5;69D4-6	++++
*Df(3L)BSC10*	69D4-5;69F5-7	++++
*Df(3L)BSC12*	69F6-70A1;70A1-2	++
*Df(3L)fz-GF3b*	70C1-2;70D4-5	+++
*Df(3L)XG5*	71C2-3;72B1-C1	++
*Df(3L)fz2*	75F10-11;76A1-5	++++
*Df(3L)ME107*	77F3;78C8-9	+++
*Df(3R)ME15*	81F3-6;82F5-7	++
*Df(3R)3-4*	82F3-4;82F10-11	++++
*Df(3R)p-XT103*	85A2;85C1-2	++++
*Df(3R)BSC38*	85F1-2;86C7-8	++
*Df(3R)sbd105*	88F9-89A1;89B9-10	++
*Df(3R)sbd104*	89B5;89C2-7	++
*Df(3R)P115*	89B7-8;89E7	+++
*Df(3R)Exel9012*	94E9;94E13	+++
*Df(3R)Exel6195*	95A4;95B1	++
*Df(3R)Exel9014*	95B1;95D1	+++
*Df(3R)Exel6196*	95C12;95D8	+++
*Df(3R)crb-F89-4*	95D7-D11;95F15	+++
*Df(3R)slo8*	96A2-7;96D2-4	++++
*Df(3R)Exel6202*	96C9;96E2	+++
*Df(3R)Exel6203*	96E2;96E6	++++
*Df(3R)B81*	99D3;3Rt	++++

^a ^Each '+' represents 0.5 SD from the mean (~22.4%) observed for *park RNAi *alone flies.

**Table 3:** List of *deficiencies *showing lethal interactions with *park *knockdown

*Deficiencies*	Breakpoints
*Df(2L)BSC16*	21C3-4;21C6-8
*Df(2L)ast2*	21D1-2;22B2-3
*Df(2L)BSC37*	22D2-3;22F1-2
*Df(2L)dpp[d14]*	22E4-F2;22F3-23A1
*Df(2L)C144*	22F4-23A1;23C2-4
*Df(2L)Exel6011*	25C8;25D5
*Df(2L)N22-14*	29C1-2;30C8-9
*Df(2L)J2*	31B;32A
*Df(2L)cact-255rv64*	35F-36A;36D
*Df(2L)TW137*	36C2-4;37B9-C1
*In(2R)bw[VDe2L]Cy[R]*	h42-h43;42A2-3
*Df(2R)Np5*	44F10;45D9-E1
*Df(2R)BSC29*	45D3-4;45F2-6
*Df(2R)X1*	46C;47A1
*Df(2R)en-A*	47D3;48B2
*Df(2R)vg-C*	49A4-13;49E7-F1
*Df(2R)CX1*	49C1-4;50C23-D2
*Df(2R)BSC18*	50D1;50D2-7
*Df(2R)Exel7130*	50D4;50E4
*Df(2R)Jp1*	51D3-8;52F5-9
*Df(2R)BSC49*	53D9-E1;54B5-10
*Df(2R)PC4*	55A;55F
*Df(2R)Egfr5*	57D2-8;58D1
*Df(2R)ED4065*	60C8;60E8
*Df(2R)Kr10*	60F1;60F5
*Df(3L)M21*	62F;63D
*Df(3L)HR119*	63C2;63F7
*Df(3L)GN34*	63E6-9;64A8-9
*Df(3L)ZN47*	64C;65C
*Df(3L)vin5*	68A2-3;69A1-3
*Df(3L)st-f13*	72C1-D1;73A3-4
*Df(3L)81k19*	73A3;74F
*Df(3L)W10*	75A6-7;75C1-2
*Df(3L)ED4978*	78D5;79A2
*Df(3L)BSC223*	79A3;79B3
*Df(3R)Exel6144*	83A6;83B6
*Df(3R)WIN11*	83E1-2;84A5
*Df(3R)p712*	84D4-6;85B6
*Df(3R)M-Kx1*	86C1;87B1-5
*Df(3R)T-32*	86E2-4;87C6-7
*Df(3R)ry615*	87B11-13;87E8-11
*Df(3R)ea*	88E7-13;89A1
*Df(3R)DG2*	89E1-F4;91B1-B2
*Df(3R)Dl-BX12*	91F1-2;92D3-6
*Df(3R)e-R1*	93B6-7;93D2
*Df(3R)Exel6197*	95D8;95E5
*Df(3R)Espl3*	96F1;97B1
*Df(3R)IR16*	97F1-2;98A
*Df(2L)BSC16*	21C3-4;21C6-8
*Df(2L)ast2*	21D1-2;22B2-3
*Df(2L)BSC37*	22D2-3;22F1-2

Above *deficiencies*, when crossed into the *park *knockdown background, significantly reduced the viability of *park *knockdown flies (less than five flies eclosed).

### F1 screen for modifiers of the *Pink1 *knockdown phenotype

Above *deficiencies *were also screened using the *Pink1 *knockdown mutant background. *Pink1 *knockdown mutant flies displayed the wing-posture phenotype at the penetrance of ~64% (n = 314) at 25°C. Among 26 enhancer-containing cytological regions identified from the *Park *screen (Table 1), 8 cytological regions, when reduced by 50% in dosage, also enhanced the penetrance of the *Pink1 *knockdown wing phenotype (Table 4). This screen also identified 9 enhancer-containing cytological regions that were not uncovered from the *park *screen (Table 4). Among 53 suppressor-containing cytological regions identified from the *park *screen (Table 2), we found that 23 cytological regions also contained suppressors of the *Pink1 *wing-posture phenotype (Table 5). In addition, we found that 30 cytological regions, when reduced by 50% in dosage, suppressed the *Pink1 *wing phenotype but not the *park *wing phenotype (Table 5).

**Table 4:** Enhancers of the *Pink1-RNAi *wing phenotype

*Deficiencies*	Breakpoints	**Strength of modification**^**a**^
***Df(2L)net-PMF***	**21A1;21B7-8**	++
*Df(2L)BSC4*	21B7-C1;21C2-3	++
*Df(2L)BSC16*	21C3-4;21C6-8	++
***Df(2L)BSC17***	**30C3-5;30F1**	++
***Df(2L)BSC50***	**30F5;31B1**	+++
***Df(2R)nap9***	**42A1-2;42E6-F1**	++
***Df(2R)cn9***	**42E;44C**	++
*Df(2R)BSC29*	45D3-4;45F2-6	++
***Df(2R)BSC39***	**48C5-D1;48D5-E1**	++
*Df(2R)BSC3*	48E12-F4;49A11-B6	+++
*Df(2R)BSC22*	56D7-E3;56F9-12	++
*Df(3L)BSC27*	65D4-5;65E4-6	++
*Df(3L)BSC14*	67E3-7;68A2-6	+++
*Df(3L)XG5*	71C2-3;72B1-C1	+++
*Df(3L)ED4782*	75F2;76A1	++
*Df(3L)HD1*	79D3-E1;79F3-6	++
***Df(3R)BSC47***	**83B7-C1;83C6-D1**	++
***Df(3R)Tpl10***	**83C1-2;84B1-2**	++

Each *deficiency *was crossed into the *Pink1 *knockdown background and the wing posture phenotype was scored. Crosses were maintained at 25°C.

^a ^Each '+' represents 1.0 SD from the mean (i.e. ~64.5%) observed for *Pink1 RNAi *alone flies. *Deficiencies *that also enhanced *park RNAi *wing posture phenotype (Table 1) are indicated in bold.

**Table 5:** Suppressors of the *Pink1-RNAi *wing phenotype

*Deficiencies*	Breakpoints	**Strength of modification**^**a**^
***Df(2L)BSC106***	**21B7;21C2**	+++
***Df(2L)dp-79b***	**22A2-3;22D5-E1**	+++++
*Df(2L)JS17*	23C1-2;23E1-2	+++
*Df(2L)drm-P2*	23F3-4;24A1-2	+++
***Df(2L)ed1***	**24A2;24D4**	+++
***Df(2L)BSC109***	**25C4;25C8**	++++
***Df(2L)E110***	**25F3-26A1;26D3-11**	++++
*Df(2L)BSC6*	26D3-E1;26F4-7	++++
*Df(2L)Dwee1-W05*	27C2-3;27C4-5	+++
*Df(2L)XE-3801*	27E2;28D1	+++
***Df(2L)BSC142***	**28C3;28D3**	++++
***Df(2L)BSC143***	**31B1;31D9**	+++
*Df(2L)BSC32*	32A1-2;32C5-D1	+++++
*Df(2L)BSC147*	34C1;34C6	++++
*Df(2L)Exel6049*	40A5;40D3	+++
*Df(2R)w45-30n*	45A6-7;45E2-3	++++
*Df(2R)CB21*	48E;49A	+++
*Df(2R)Exel7130*	50D4;50E4	++++
***Df(2R)Exel7131***	**50E4;50F6**	+++++
*Df(2R)BSC11*	50E6-F1;51E2-4	+++
***Df(2R)BSC550***	**53C1;53C6**	++++
***Df(2R)robl-c***	**54B17-C4;54C1-4**	+++
*Df(2R)k10408*	54C1-4;54C1-4	+++
***Df(2R)P34***	**55E2-4;56C1-11**	++++
*Df(2R)Exel7162*	56F11;56F16	+++
*Df(2R)or-BR6*	59D5-10;60B3-8	+++
*Df(3L)M21*	62F;63D	+++
*Df(3L)GN34*	63E6-9;64A8-9	+++
***Df(3L)XDI98***	**65A2;65E1**	+++
***Df(3L)BSC33***	**65E10-F1;65F2-6**	+++
***Df(3L)66C-G28***	**66B8-9;66C9-10**	+++
*Df(3L)BSC13*	66B12-C1;66D2-4	+++
***Df(3L)Scf-R6***	**66E1-6;66F1-6**	+++++
***Df(3L)BSC10***	**69D4-5;69F5-7**	+++
*Df(3L)st-f13*	72C1-D1;73A3-4	++++
*Df(3L)81k19*	73A3;74F	+++++
*Df(3L)kto2*	76B1-2;76D5	+++
*Df(3L)ri-79c*	77B-C;77F-78A	++++
*Df(3L)ri-XT1*	77E2-4;78A2-4	++++
***Df(3L)ME107***	**77F3;78C8-9**	+++
*Df(3L)BSC249*	79B2;79D2	++++
***Df(3R)p-XT103***	**85A2;85C1-2**	+++++
*Df(3R)M-Kx1*	86C1;87B1-5	++++
*Df(3R)ea*	88E7-13;89A1	+++
***Df(3R)sbd104***	**89B5;89C2-7**	+++
***Df(3R)P115***	**89B7-8;89E7**	++++
*Df(3R)23D1*	94A3-4;94D1-4	+++
***Df(3R)crb-F89-4***	**95D7-D11;95F15**	+++
*Df(3R)Exel6197*	95D8;95E5	+++
***Df(3R)Exel6202***	**96C9;96E2**	+++
***Df(3R)Exel6203***	**96E2;96E6**	++++
*Df(3R)Tl-P*	97A;98A1-2	+++
*Df(3R)IR16*	97F1-2;98A	+++++

^a ^Each '+' represents 1.0 SD from the mean (i.e. ~64.5%) observed for *Pink1 RNAi *alone flies. *Deficiencies *that also suppressed the *park RNAi *wing posture phenotype (Table 2) are indicated in bold.

Among 50 cytological regions that caused adult lethality when their dosage was reduced by 50% in *park *knockdown background (Table 3), 21 cytological regions also displayed a similar lethal interaction with *Pink1 *knockdown (Table 6). Five cytological regions only showed lethal interactions with *Pink1 *but not *park *(Table 6).

**Table 6:** List of *deficiencies *showing lethal interactions with *Pink1 *knockdown

*Deficiencies*	Breakpoints
***Df(2L)BSC37***	**22D2-3;22F1-2**
***Df(2L)dpp[d14]***	**22E4-F2;22F3-23A1**
***Df(2L)C144***	**22F4-23A1;23C2-4**
*Df(2L)sc19-8*	24C2-8;25C8-9
***Df(2L)Exel6011***	**25C8;25D5**
*Df(2L)b87e25*	34B12-C1;35B10-C1
***Df(2L)TW137***	**36C2-4;37B9-C1**
***In(2R)bw[VDe2L]Cy[R]***	**h42-h43;42A2-3**
*Df(2R)M41A4*	41A;41A
***Df(2R)X1***	**46C;47A1**
***Df(2R)CX1***	**49C1-4;50C23-D2**
***Df(2R)BSC49***	**53D9-E1;54B5-10**
***Df(2R)ED4065***	**60C8;60E8**
***Df(2R)Kr10***	**60F1;60F5**
***Df(3L)HR119***	**63C2;63F7**
***Df(3L)vin5***	**68A2-3;69A1-3**
*Df(3L)vin7*	68C8-11;69B4-5
***Df(3L)W10***	**75A6-7;75C1-2**
***Df(3L)ED4978***	**78D5;79A2**
***Df(3L)BSC223***	**79A3;79B3**
***Df(3R)Exel6144***	**83A6;83B6**
***Df(3R)p712***	**84D4-6;85B6**
***Df(3R)T-32***	**86E2-4;87C6-7**
***Df(3R)DG2***	**89E1-F4;91B1-B2**
***Df(3R)Dl-BX12***	**91F1-2;92D3-6**
*Df(3R)B81*	99D3;3Rt

Above *deficiencies*, when crossed into the *Pink1 *knockdown background, significantly reduced the viability of *Pink1 *knockdown flies (less than five flies eclosed). *Deficiencies *that display a similar lethal interaction with *park *knockdown (Table 3) are indicated in bold.

### Analysis of genetic interactions using a *Pink1 *null mutant allele

Cytological regions identified from above *RNAi*-based screen may contain genes that function in the *Pink1/park *pathway, or genes that function in a parallel pathway that act together with the *Pink1/park *pathway to regulate mitochondrial function. To further characterize these cytological regions, we performed genetic analysis using a *Pink1 *null mutant allele to test the potential interactions between *Pink1 *and cytological regions that interact with both *Pink1 *and *park *in the above *RNAi*-based screen. Among six enhancer-containing cytological regions examined, five cytological regions, when reduced by 50% in dosage, also enhanced the wing phenotype in *Pink1 *null mutants (Table 7). Among 17 suppressor-containing cytological regions examined, 10 cytological regions, when reduced by 50% in dosage, also suppressed the wing phenotype in *Pink1 *null mutants (Table 7). Among 19 examined cytological regions that showed lethal interactions with both *Pink1 *and *park *in *RNAi*-based screens, 5 cytological regions, when reduced by 50% in dosage, also displayed the lethal phenotype in *Pink1 *null mutants (Table 8).

**Table 7:** Analysis of the interaction between a *Pink1 *null mutation and cytological regions that modified both *park-RNAi *and *pink1-RNAi *wing phenotype

		Effects of modification		
		
*Deficiencies*	Breakpoints	*Pink1-RNAi*	*park-RNAi*	***Pink1***^***B9***^
*Enhancers*				
*Df(2L)net-PMF*	21A1;21B7-8	++	++	n/d
*Df(2L)BSC17*	30C3-5;30F1	++	++	n/d
*Df(2L)BSC50*	30F5;31B1	+++	++++++	En
*Df(2R)nap9*	42A1-2;42E6-F1	++	+++++	En
*Df(2R)cn9*	42E;44C	++	++	En
*Df(2R)BSC39*	48C5-D1;48D5-E1	++	++++	En
*Df(3R)BSC47*	83B7-C1;83C6-D1	++	++	En
*Df(3R)Tpl10*	83C1-2;84B1-2	++	++	No
				
*Suppressors*				
*Df(2L)BSC106*	21B7;21C2	---	----	Su
*Df(2L)dp-79b*	22A2-3;22D5-E1	-----	----	No
*Df(2L)ed1*	24A2;24D4	---	----	n/d
*Df(2L)BSC109*	25C4;25C8	----	----	Su
*Df(2L)E110*	25F3-26A1;26D3-11	----	--	n/d
*Df(2L)BSC142*	28C3;28D3	----	----	Su
*Df(2L)BSC143*	31B1;31D9	---	--	No
*Df(2R)Exel7131*	50E4;50F6	-----	----	Su
*Df(2R)BSC550*	53C1;53C6	----	--	No
*Df(2R)robl-c*	54B17-C4;54C1-4	---	--	n/d
*Df(2R)P34*	55E2-4;56C1-11	----	----	Su
*Df(3L)XDI98*	65A2;65E1	---	--	n/d
*Df(3L)BSC33*	65E10-F1;65F2-6	---	--	n/d
*Df(3L)66C-G28*	66B8-9;66C9-10	---	---	No
*Df(3L)Scf-R6*	66E1-6;66F1-6	-----	--	Su
*Df(3L)BSC10*	69D4-5;69F5-7	---	----	Su
*Df(3L)ME107*	77F3;78C8-9	---	---	No
*Df(3R)p-XT103*	85A2;85C1-2	-----	----	Su
*Df(3R)sbd104*	89B5;89C2-7	---	--	n/d
*Df(3R)P115*	89B7-8;89E7	----	---	Su
*Df(3R)crb-F89-4*	95D7-D11;95F15	---	---	No
*Df(3R)Exel6202*	96C9;96E2	---	---	No
*Df(3R)Exel6203*	96E2;96E6	----	----	Su
				

Abbreviations: n/d, not determined; Su, suppression; En, enhancement; No, no modification.

**Table 8:** Analysis of the interaction between a *Pink1 *null allele and *deficiencies *that exhibited lethal interactions with both *park *and *Pink1 *knockdown

*Deficiencies*	Breakpoints	**Synthetic lethal with *pink1***^**B9**^
*Df(2L)BSC37*	22D2-3; 22F1-2	No
*Df(2L)dpp[d14]*	22E4-F2; 22F3-23A1	Yes
*Df(2L)C144*	22F4-23A1; 23C2-4	Partial
*Df(2L)Exel6011*	25C8; 25D5	No
*Df(2L)TW137*	36C2-4; 37B9-C1	No
*In(2R)bw[VDe2L]Cy[R]*	h42-h43;42A2-3	Yes
*Df(2R)M41A4*	h44;42A2	Yes
*Df(2R)X1*	46C;47A1	n/d
*Df(2R)CX1*	49C1-4;50C23-D2	Yes
*Df(2R)BSC49*	53D9-E1;54B5-10	No
*Df(2R)ED4065*	60C8;60E8	
*Df(2R)Kr10*	60F1;60F5	No
*Df(3L)HR119*	63C2;63F7	No
*Df(3L)vin5*	68A2-3;69A1-3	No
*Df(3L)vin7*	68C8-11;69B4-5	No
*Df(3L)W10*	75A6-7;75C1-2	No
*Df(3L)ED4978*	78D5;79A2	No
*Df(3L)BSC223*	79A3;79B3	No
*Df(3R)Exel6144*	83A6;83B6	No
*Df(3R)p712*	84D4-6;85B6	n/d
*Df(3R)T-32*	86E2-4;87C6-7	No
*Df(3R)Dl-BX12*	91F1-2;92D3-6	No

### Molecular characterization of the PD-interacting cytological region 21A1-21B7

The PD-interacting cytological regions identified from above screens are relative large and contain a number of genes. As a first step towards molecular characterization of these PD-interacting cytological regions, we performed fine mapping in four selected PD-interacting cytological regions to identify corresponding PD-interacting genes. Those cytological regions were selected since they displayed strongest interactions with both *park *and *Pink1*.

**Figure 3:**
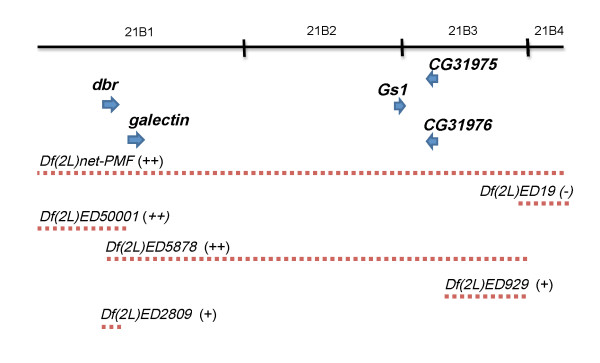
**Molecular characterization of the PD-interacting cytological region 21A1-21B7**. Genes (arrows) are listed according to their genomic location. The regions uncovered by the *deficiencies *used in the experiments are indicated (dashed lines). The effect of each *deficiency *is indicated as enhancement (+) or no enhancement (-).

From above screens, we found that reducing the dosage of the cytological region 21A1-21B7-8, deleted in the *deficiency *chromosome *Df(2L) net-PMF*, enhanced both *park *and *Pink1 *wing phenotype (Table 1 and 4). To identify the corresponding PD-interacting gene within this cytological region, we tested additional *deficiency *lines that carry smaller deletions within this region. We found that similar enhancement was observed when a smaller *deficiency *chromosome *Df(2L)ED5878 *was crossed into *park *or *Pink1 *knockdown background (Figure 3). Twenty two genes are disrupted in this *deficiency *chromosome, including *dbr, galectin, CG11374, net, CG11376, Sam-S, CG4822, Gs1, CG31976, CG3709, CG11377, CG13694, CG31975, CG11455, Nhe1, CG3164, CG31974, CG3436, CG11454, CG33635, CG42399 *and *spen*. Interestingly, we found that another smaller *deficiency Df(2L) ED2809 *in which only the *debra *(*dbr*) gene is deleted, also enhanced the *park *knockdown phenotype (~50% increase in penetrance compared to *park RNAi *alone, n = 104). Taken together, these results suggest strongly that *dbr *is largely, if not entirely, responsible for the observed interaction with PD genes.

### Molecular characterization of two PD suppressor-containing cytological regions 21B7-21C2 and 50E4-50F6

**Figure 4:**
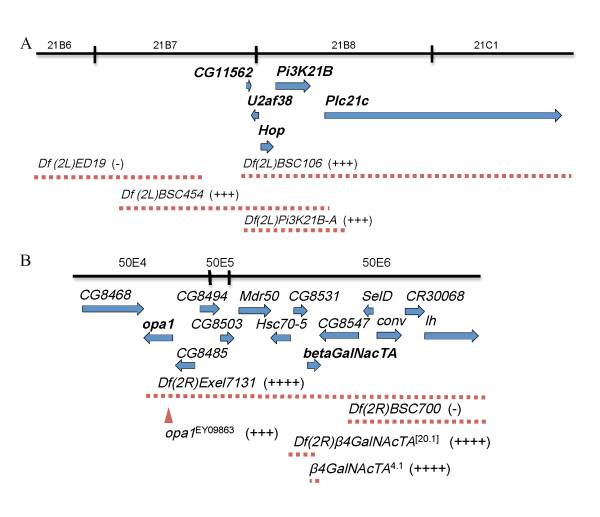
**Molecular characterization of two PD suppressor-containing cytological regions 21B7-21C2 and 50E4-50F6**. A, Characterization of the PD-interacting cytological region 21B7-21C2. B, Characterization of the PD-interacting cytological region 50E4-50F6. The regions uncovered by the *deficiencies *used in the experiments are indicated (dashed line). The effect of each *deficiency *is indicated as suppression (+) or no suppression (-). The genes (arrows) are listed according to their genomic location. *opa1*^EY09863 ^is an *opal *loss-of-function allele in which a P-element-containing sequence is inserted into the *opa1 *locus. *Df(2R)β4GalNAcTA*^[20.1] ^and *β4GalNAcTA*^4.1 ^were generated by imprecise excision [47]. In the *Df(2R)β4GalNAcTA*^[20.1] ^allele, both *β4GalNAcTA *and CG8531 are deleted, while 610 base-pair sequence in the *β4GalNAcTA *gene is deleted in the *β4GalNAcTA*^4.1 ^allele.

Reducing the dosage of the cytological region 21B7-21C2, uncovered by the *deficiency *chromosome *Df(2L)BSC106*, suppressed both *park *and *Pink1 *wing phenotype (Table 2 and 5). From a collection of smaller *deficiencies *mapped within this region, we identified two overlapping *deficiencies Df(2L)BSC454 *and *Df(2L)Pi3K21B*, which like *Df(2L)BSC106*, both suppressed *park *and *Pink1 *wing phenotype (Figure 4A). The cytological region deleted in both *Df(2L)BSC454 *and *Df(2L)Pi3K21B*, contains four genes *Hop, Pi3K21B, Plc21C and U2af38*.

To further narrow down the PD-interacting gene within this region, we tested if any of above four genes interacts with PD genes. Among them, we found that knockdown the expression of *Pi3K21B *also significantly suppressed the *Pink1 *wing phenotype (~48% decrease in penetrance compared to *Pink1 RNAi *alone, n = 42). This result suggests that *Pi3K21B *is the corresponding PD-interacting gene.

Reducing the dosage of the cytological region 50E4-50F6, uncovered by the *deficiency *chromosome *Df(2R)Exel7131*, also suppressed both *park *and *Pink1 *knockdown wing phenotype (Table 2 and 5). However, another *deficiency Df(2R)BSC700*, in which the deleted cytological region partially overlaps with that affected in *Df(2R)Exel7131*, did not interact with *park *or *Pink1*. The cytological region deleted in *Df(2R)Exel7131*, but not in *Df(2R)BSC700*, carry nine genes (i.e. *opa1-like, CG8485, CG8494, CG8503, Mdr50, Hsc70-5, CG8531, β4GalNAcTA and CG8547*) (Figure 4B).

To test if the above genes interact with *park *or *Pink1*, we crossed available mutations into *park *or *Pink1 *knockdown background. We found that *opa1 *and *β4GalNAcTA *interact genetically with PD genes (Figure 4B). A heterozygous mutation of *opa1 *(i.e. *opa1*^EY09863^) significantly suppressed the *park *wing phenotype (~95% reduction in penetrance compared to *park *knockdown alone, n = 83). And heterozygous mutations of *β4GalNAcTA*, *Df (2R)β4GalNAcTA*^[20.1] ^(deleting both *β4GalNAcTA *and its neighboring gene *CG8547*) and *β4GalNAcTA*^4.1 ^(deleting part of the *β4GalNAcTA *gene only), significantly suppressed the *Pink1 *wing phenotype (for *Df (2R)β4GalNAcTA*^[20.1]^, ~92% reduction in penetrance compared to *Pink1 *knockdown alone, n = 62; for *β4GalNAcTA*^4.1^, ~82% reduction in penetrance compared to *Pink1 *knockdown alone, n = 59).

### *drp1 *is the corresponding gene of the cytological region 22F4-23A3 that displayed lethal interaction with PD genes

Two *deficiencies, Df(2L)dpp*^[d14] ^(22E4-F2;22F3-23A1) and *Df(2L)C144 *(22F4-23A1;23C2-4), caused lethality when heterozygous in *park RNAi*, *Pink1 RNAi *or *Pink1 *null mutant background (Table 8). A smaller *deficiency *(i.e. *Df(2L)ED136)*, which deletes the overlapping region defined by the above *deficiencies*, also caused partial lethality in the *Pink1 *null background (i.e. ~50% reduction in viability compared to controls). The cytological region deleted in *Df(2L)ED136 *contains 29 genes, of which mutations in *drp1 *have been previously implicated as an enhancer of *park *and *Pink1 *mutant phenotypes [16,33,34]. Hence, we used a mutant allele for *drp1 *(i.e. *drp*1^KG03815^) to examine the potential interaction. Consistent with previous reports, we found that *drp1 *heterozygosity substantially enhanced the lethal phenotype in the *Pink1 *null background (i.e. ~82.8% reduction in viability compared to controls). This result strongly suggests that *drp1 *is the corresponding gene within the cytological region 22F4-23A3 that displayed lethal interaction with PD genes.

## Discussion

In this study, we performed a genome-wide screen to isolate modifiers of PD genes. From this screen, we identified a number of cytological regions that interact with *park *and/or *Pink1*. Fine mapping of selected PD-interacting cytological regions led to the identification of corresponding PD-interacting genes. Among them, *opa1 *and *drp1 *have previously been implicated in *Pink1/park*-mediated mitochondrial quality control pathways. In addition, we also identified *debra*, *Pi3K21B*, and *β4GalNAcTA *as novel PD-interacting genes.

While several previous studies suggest that *park *and *Pink1 *function in a common pathway to regulate mitochondrial function, cytological regions identified from our *park*- and *Pink1*-modifying screens do not completely overlap. For instance, among cytological regions showing lethal interactions with *Pink1*, about 81% displayed similar interactions with *park *(Table 6). Among cytological regions modifying *Pink1 *wing phenotype, only ~44% showed similar interactions with *park *(Table 4 and 5). One possible explanation is that *park *and *Pink1 *knockdown genetic background have different sensitivity, which may account for the difference in their interactions with some cytological regions. Alternatively or additionally, the molecular network involving Park and Pink1 may be more complex than a simplified linear pathway.

A previous study by Pallanck and colleagues screened a collection of P-element insertions (covering less than 10% of the fly genome) that modify the partial lethality of *park *null mutants [30]. However, since their screen was conducted in homozygous *park *null mutant background, less than 10% of the fly genome was covered. To increase the coverage, we developed an *RNAi*-based strategy, which allowed us to perform a F1 screen that covered >80% of the fly genome. Several PD-interacting genes identified by Pallanck and colleagues in their previous screen [30], are located within PD-interacting cytological regions identified from our screen. For instance, *Glutathione S-transferase *1 (*Gst1*) and *Thioredoxin-2 (Trx-2) *are located in PD-interacting cytological regions uncovered by *Df(2R)BSC49 *(Table 6) and *Df(2L)N22-14 *(Table 3), respectively.

While our screen using *deficiencies *greatly increases the coverage of genomic regions, there are several limitations. For instance, since cytological regions deleted in *deficiency *chromosomes contain a large number of genes (average ~50), it is possible that a cytological region containing PD-interacting genes may not be identified from our screen if both enhancers and suppressors are located within the same region. Similarly, this may also make it difficult to identify the corresponding genes, especially if the strong modifying effect is due to the presence of multiple weak modifiers within the same region. Additionally, since those *deficiency *chromosomes used in our screen may carry second-site mutations contributing to the observed interactions, it is necessary to characterize independent point mutations and/or deletions mapped within the same region.

Our screen isolated two known PD-interacting genes *drp1 *and *Opa1*. *drp1 *encodes a GTPase (i.e. the dynamin-related protein 1) that has been previously implicated in regulating mitochondrial fission [35], while *opa1 *(*optic atrophy 1*) encodes for another dynamin-related GTPase that promotes mitochondrial fusion [36,37]. Consistent with previous reports [16,33,34], we showed that *drp1 *heterozygosity induced lethality prior to the adult stage in *park *or *Pink1 *knockdown background. We also showed that *opa1 *heterozygosity significantly suppressed the *park-RNAi*-induced wing phenotype. Similarly, previous reports showed that heterozygous mutations of *opa1 *suppressed indirect flight muscle degeneration and mitochondrial morphological phenotypes in *Pink1 *and *park *mutants [33,34]. Together, these observations underscore the importance of PD-interacting genes in mitochondrial fission and fusion to facilitate mitochondrial quality control.

Among the three novel PD-interacting genes (i.e. *debra*, *Pi3K21B*, and *β4GalNAcTA*) isolated from our screen, *debra *(*de*terminer of *br*eaking down of Ci *a*ctivator) (*dbr*) heterozygosity led to strong enhancement of the *park*-*RNAi-*induced wing phenotype. *dbr *encodes a novel zinc-binding protein of 1007 amino-acid residues [38]. Cell culture studies showed that Dbr forms a complex with Slimb, a component of the SCF (Skpl, Cdc53 and F box) ubiquitin ligase complex, to mediate the polyubiquitination of the transcription factor Cubitus interruptus (Ci) and thus targets Ci into the lysosome for degradation [38]. This raises the interesting possibility that Dbr functions together with Park in the ubiquitin-proteasome pathway for the control of protein quality. Reducing the dosage of *dbr *may thus increase the accumulation of toxic protein substrates, leading to the enhancement of the *park *phenotype. In this context, it is worth noting that a recent study showed that reducing the level of *dbr *also enhanced *Ataxin3-*induced neurodegeneration in *Drosophila*, which also resulted from accumulation of pathogenic proteins [39]. Additionally, since Dbr is a zinc-binding protein, Dbr may also play a role in regulating the level of intracellular zinc. Zinc dyshomeostasis has been shown to cause abnormalities in autophagy that are associated with Alzheimer's disease, Parkinson's disease, and Huntington's disease [40]. Thus, it is possible that in addition to its interaction with Park in the ubiquitin-proteasome pathway, Dbr may interact with the PD pathway by regulating autophagy.

Another novel-PD-interacting gene *Pi3K21B*, identified in our screen as a suppressor of PD wing phenotype, encodes an SH2 domain-containing adaptor protein that binds to the *Drosophila *class IA Phosphoinisitide 3 Kinase (PI3K), Pi3K92E/Dp110 [41]. It has been shown that class IA PI3-kinases are activated by nutrient-responsive insulin signalling to regulate cell growth and proliferation [42]. Loss of Pi3K21B-binding sites completely abrogates the activation of Dp110 by the insulin receptor, which decreased cell growth leading to reduced body size [43]. One possible explanation for the observed interaction between *Pi3K21B *and PD genes is that reducing the level of *Pi3K21B *may decrease insulin signaling and metabolic activities. This may be achieved by reducing the level of the TOR (target of rapamycin) signaling pathway. TOR can be activated by the PI3K/Akt pathway to regulate cell growth and metabolism (for review, see [44]). Recent studies show that reducing TOR signaling rescued PD phenotypes in *Drosophila *by decreasing S6 kinase-mediated 5'-Cap-dependent translation [45], and increasing 4E-BP-promoted 5'-Cap-independent translation [46]. Similarly, we speculate that *Pi3K21B *heterozygosity promotes 5'-Cap-independent translation by reducing TOR signaling, thus increasing the production of pro-survival factors leading to the suppression of PD phenotypes.

Characterization of the suppressors of the *Pink1-RNAi-*induced wing phenotype also identified *β4GalNAcTA *as a novel PD-interacting gene. *β4GalNAcTA *encodes for a β 1,4-N acetlygalactosaminyltransferase that mediates the N-glycosylation of protein substrates [47]. *Drosophila *adult mutants of *β4GalNAcTA *display severe locomotion abnormalities such as a low climbing index and coordination defects [48]. Glycosylation may affect protein function by diverse mechanisms, such as promoting protein stability, enabling protein recognition, altering ligand affinity and inhibiting protein activity [49]. For instance, abnormal glycosylation of alpha-dystroglycan interferes with its function leading to congenital muscular dystrophy [50]. Glycosylation may also contribute to the misfolding and accumulation of several proteins implicated in neurodegenerative disorders. For instance, glycosylation of tau and amyloid precursor protein (APP) may promote the formation and accumulation of pathogenic advanced glycosylation end-products (AGEs) [51]. In addition, α-synuclein, the primary component of Lewy bodies in Parkinson's disease, is also modified by glycosylation [9]. This modification is hypothesized to affect the clearance of α-synuclein aggregates [9]. We speculate that glycosylation mediated by *β4GalNAcTA *affects the stability and/or activity of components in the PD pathways, which may contribute to the accumulation of toxic proteins, increased sensitivity to oxidative damage and mitochondrial dysfunction. Future studies will be needed to elucidate the exact action of *β4GalNAcTA *in the PD pathways.

## Conclusion

Systematic genetic screens covering ~80% of the entire genome were performed to identify modifiers of the PD phenotype in *Drosophila*. From the screen, we identified a number of cytological regions that interact with *park *and/or *Pink1*. Fine mapping in selected PD-interacting cytological regions was performed, which identified *debra, Pi3K21B *and *β4GalNAcTA *as novel PD-interacting genes. Future characterization of other PD-interacting cytological regions will likely lead to the identification of additional PD-interacting genes.

## Competing interests

The authors declare that they have no competing interests.

## Authors' contributions

CF conducted all experiments, and was involved in writing the manuscript. YR supervised and wrote the manuscript. All authors read and approve the manuscript.
